# Antibiotic Resistance in Fermented Foods Chain: Evaluating the Risks of Emergence of *Enterococci* as an Emerging Pathogen in Raw Milk Cheese

**DOI:** 10.1155/ijm/2409270

**Published:** 2024-12-26

**Authors:** Celso Raul Silambo Chaves, Acácio Salamandane, Emília Joana F. Vieira, Cátia Salamandane

**Affiliations:** ^1^Clinical Laboratory of the Matacuane Military Health Center, Avenida Alfredo Lawley No 42, Matacuane, Beira, Mozambique; ^2^Department of Nutrition, Faculty of Health Sciences, Lúrio University, Marrere Campus, Nampula 4250, Mozambique; ^3^Laboratory of Active Principles, National Center for Scientific Research, Ministry of Higher Education, Science, Technology and Innovation, Avenida Ho Chi Min No 201, Luanda, Angola; ^4^Laboratory of Food Quality and Safety, Lúrio Interdisciplinary Research Center, Lúrio University, Marrere Campus, Nampula 4250, Mozambique

**Keywords:** antibiotic resistance genes, cross-contamination, food safety, food supply chain, lactic acid bacteria, raw milk cheese

## Abstract

Fermented foods, particularly fermented dairy products, offer significant health benefits but also present serious concerns. Probiotic bacteria, such as lactic acid bacteria (LAB), found in these foods have been strongly linked to the selection and dissemination of antibiotic resistance genes (ARGs). This study aims to examine the potential risks associated with fermented foods, despite their importance in human nutrition, by analyzing the entire production chain from raw material acquisition to storage. Focusing on cheese production as a key fermented food, the study will investigate various aspects, including dairy farm management, milk acquisition, milk handling, and the application of good manufacturing practices (GMP) and good hygiene practices (GHP) in cheese production. The findings of this review highlight that ARGs found in LAB are similar to those observed in hygiene indicator bacteria like *E. coli* and pathogens like *S. aureus*. The deliberate use of antibiotics in dairy farms and the incorrect use of disinfectants in cheese factories contribute to the prevalence of antibiotic-resistant bacteria in cheeses. Cheese factories, with their high frequency of horizontal gene transfer, are environments where the microbiological diversity of raw milk can enhance ARG transfer. The interaction between the raw milk microbiota and other environmental microbiotas, facilitated by cross-contamination, increases metabolic communication between bacteria, further promoting ARG transfer. Understanding these bacterial and ARG interactions is crucial to ensure food safety for consumers.

## 1. Introduction

The overutilization of antibiotics in dairy cattle on livestock farms may lead to the emergence of antibiotic-resistant pathogens within the food supply chain, particularly within raw milk processing factories [1, 2]. The dairy food supply chain facilitates the transmission of antibiotic-resistant bacteria (ARB) between animals and humans, particularly through the use of raw milk in fermented products like traditional raw milk cheese [3–5]. In recent years, the approach of foodborne pathogens has expanded beyond on virulence factors to encompass an investigation into the presence of antibiotic resistance genes (ARGs), carried by both pathogens and commensal bacteria found in food [2, 6]. The potential of foodborne bacteria to harbor antibiotic resistance determinants raises concerns about their role in spreading resistance [5, 7]. This is especially worrisome if these bacteria become opportunistic pathogens, even more when resistance genes are transferred to pathogenic bacteria, thereby undermining the effectiveness of antibiotics for treating common infections [8].

Fermented foods are widely acknowledged as rich sources of functional compounds that play a crucial role in nutrition and health. Such foods offer various benefits, such as reducing the risk of heart disease and promoting digestion, immunity, and weight loss [9, 10]. Several studies have focused on identifying bioactive peptides and microbial metabolites in fermented foods, strengthening the connection between these foods and their beneficial health effects [11–13]. Ebner et al. [14] reported the identification of about 236 multifunctional peptide sequences like VYPFPGPIPN, KIEKFQSEEQQQT, VLNENLLR, and NLHLPLP in kefir. Hati, Sakure, and Mandal [15] report peptides with amino acid sequences IPP and VPP with antihypertensive and antioxidative activity in *Lactobacillus helveticus*–fermented honey-based milk. Peptides sequences with ACE inhibitory activity in the order Lys-Pro-Ala-Gly-Asp-Phe > Lys-Ala-Ala-Leu-Ser-Gly-Met > Lys-Lys-Ala-Ala-Met-Ala-Met > Leu-Asp-His-Val-Pro-Gly-Gly-Ala-Arg have been produced in milk fermented by *Lactobacillus helveticus* and *Lactobacillus casei* [16]. Fermented dairy products offer an ideal delivery system for introducing probiotic bacteria that are beneficial to the human gut microbiome [10, 12].

Cheese is one of the most important fermented dairy products produced and consumed by humans. Cheese production involves the use of lactic acid bacteria (LAB) as starter cultures, such as *Lactobacillus*, *Streptococcus*, *Enterococcus*, *Bifidobacterium*, and *Leuconostoc*, during the fermentation and maturation of cheese [5, 9]. *Enterococcus* is a genus of bacteria commonly found in cheese, which is crucial in maturation. Moreover, *Enterococcus* contributes significantly for a unique flavor, aroma, and texture in many artisanal cheeses [1, 4, 17–21]. Even though *Enterococcus* plays a vital role in cheesemaking, despite the contribution of *Enterococcus faecium* and *Enterococcus faecalis* to the fermentation and ripening process, the virulence factors directly associated with and the fact they act as reservoirs for antimicrobial resistance genes (ARGs) rise debates about its presence in scientist arena [4, 22–24].

This review aims to analyze the potential risk of transmission of ARGs in the cheese production chain. This approach is particularly relevant due to the importance of cheeses in the human diet and the growing contemporary interest in fermented food consumption. The focus will be on *Enterococcus* strains involved in the fermentation of artisanal cheeses.

## 2. Food Supply and Transmission of Antibiotic-Resistant Bacteria

Chemical compounds, specifically antibiotics, are commonly used to promote growth farming, improve feed efficiency, enhance reproduction, and reduce illness and death in livestock [25]. However, excessive antibiotic use in food production can contribute to the spread of ARB by creating environments that favor the survival of resistant strains [26]. In this context, the food chain is particularly vulnerable to this problem, as bacteria can be exposed to high levels of antibiotics, especially in meat and dairy production [27].

The use of antibiotics in food production also contributes to the selection of resistance determinants and the exchange of ARGs via horizontal gene transfer (HGT) [28, 29]. Gene encoding resistance to *β*-lactams antibiotics, resistance to tetracycline, and aminoglycoside are the most common ARGs found in *Escherichia coli* and *Salmonella* recovered from livestock and poultry products [30]. Several studies have highlighted that the rise in methicillin-resistant *Staphylococcus aureus* (MRSA), is intricately linked to the overuse of antibiotics within the livestock industry. It was proven that overuse of antibiotics causes a therapy inefficiency, due to the alterations in penicillin binding caused by the PBP2′ protein encoded by the mecA gene present in MRSA [30–33].

However, the promotion of ARB in food is not limited solely to the administration of antibiotics in livestock. Handling and processing also play crucial roles in this issue, presenting critical points to be considered [34]. During processing, there are significant risks of bacterial transmission, both by handlers and the equipment used. Antibiotic resistance has been identified in various tools used in the production and processing food, especially in meat and dairy-based products [35–37]. Usually, these microorganisms are present on the food equipment in the form of biofilm—a complex and highly structured aggregation of sessile bacteria, formed on biotic or abiotic surfaces, which are resistant to high concentrations of biocides [38–40]. The contamination of food with ARB and antibiotic ARGs during production, handling and processing, distribution, and sale can significantly contribute to the spread of antimicrobial resistance throughout the food chain.

*Lactobacillus* and *Enterococcus* are the fermenting bacterial genus reporting high antibiotic resistance profile and ARGs. *Lactobacillus pentosus* and *Leuconostoc pseudomesenteroides* isolated from natural fermented table olives were reported resistance to streptomycin (83%–100%), vancomycin and teicoplanin (70%–100%), trimethoprim (76%), trimethoprim/sulfamethoxazole (71%–100%), and cefuroxime (44%) (Table 1) [41]. In addition, gene encoding multidrug resistance Efflux Pump (*NorA*), penicillin resistance (*MepA*), and fluoroquinolone resistance gene (*MdeA*) were found in *Lactobacillus pentosus* and *Leuconostoc pseudomesenteroides* (Table 1). *Lactobacillus* isolated from fermented foods showed resistance to tetracycline, erythromycin, ciprofloxacin, chloramphenicol, kanamycin, ampicillin, and clindamycin (Table 1) [42], and *tetW*, *tetM*, *tetS* encoding to tetracycline resistance, gene *ermB* encoding to erythromycin resistance were founded (Table 1).

In *Lactobacillus* isolated from fermented dairy products, the most frequent antibiotic resistance profile is related to tetracycline, erythromycin, ciprofloxacin, chloramphenicol, kanamycin, ampicillin, clindamycin (Table 1), genes encoding resistance to tetracycline (*tetM*), erythromycin (*ermB*), and gene encoding an aminoglycoside-modifying enzyme (*aph (3″)-III*) were the most frequent gene found in *Lactobacillus* isolated from fermented dairy products.

## 3. Pathways of Antimicrobial Resistance in the Cheese Factory Environment

Food chains provide an excellent vehicle for spreading ARB, spoilage, and pathogenic bacteria from farm to fork. ARB-contaminating products on farms can survive on raw and undercooked produce, potentially affecting consumer health [56, 57]. Animal-based foods like meat, eggs, and milk are a major source of ARGs in the food chain [56, 58].

The use of microorganisms in fermentation can inadvertently introduce ARGs into the food chain. The European Food Safety Authority (EFSA) addressed this concern in 2012 by issuing guidelines to mitigate the risk in starter cultures [59]. However, unlike industrial fermented foods, spontaneous fermentations like kefir, kombucha, and artisanal cheeses rely on naturally occurring microorganisms from their raw materials [5, 60–62]. Raw milk's exceptional nutritional composition provides a favorable environment for the growth of a wide range of microorganisms, from beneficial to pathogenic [63].

Microbiota of milk encompass both microorganisms associated with the mammary gland and teat, as well as contaminants introduced by diseases like mastitis [5, 64]. Despite the health benefits of using raw milk in fermented food for humans [4–6], zoonotic pathogens (including *Campylobacter* spp., Shiga toxin–producing *Escherichia coli*, *Staphylococcus aureus*, *Listeria monocytogenes*, and *Salmonella enterica*) have been well documented as the most common foodborne pathogens resulting from the contamination of raw milk [63, 65–67]. The management and control of these zoonotic pathogens in livestock often involve the routine use of antibiotics to preserve the health of animals in livestock farming [68]. However, this procedure can increase the likelihood of the emergence and spread of ARGs [68, 69].

Metagenomic analysis showed a significantly higher number of ARGs in raw milk compared to pasteurized milk [70]. In an experimental study, Liu et al. demonstrated the conjugative transfer of the *blaCMY-2* gene, associated with ceftazidime resistance, from *E. coli* in raw milk to other bacterial species [70].

**Figure 1:**
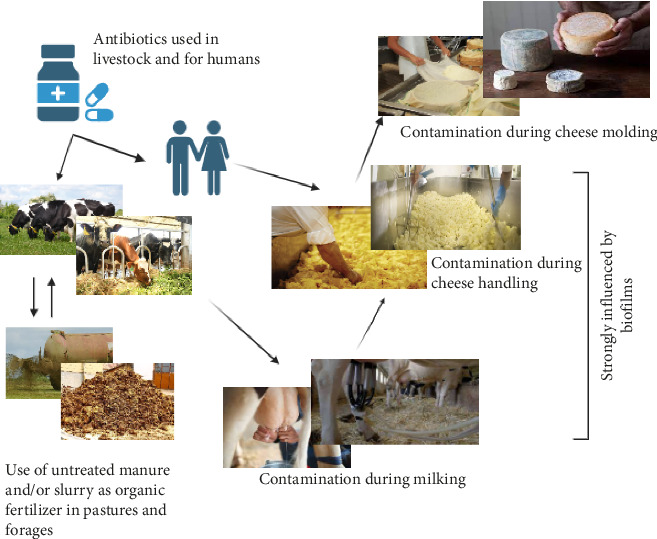
Pathways of bacterial contamination and antimicrobial resistance transmission in the cheese factory environment. This is an original figure conceptualized and created by the authors.

A largely overlooked source of ARG transmission is related to common agricultural practices, including irrigation, grazing, silage production, feed manufacturing, and the use of agricultural wastewater or animal manure (Figure 1) [5, 68]. This convergence of agricultural activities and animal waste may serve as a critical point in the dissemination of ARGs, posing a significant risk to the health of both humans and animals [71].

Irrigation plays a crucial role in agricultural production, facilitating the healthy growth of crops [72]. However, irrigation water contaminated with antibiotic residues, either from agricultural practices or environmental pollution, can contribute to the spread of these compounds and their ARGs [68, 72]. Furthermore, the frequent use of antibiotics in livestock farming and intensive agriculture contributes to the presence of these compounds in water, creating a conducive environment for the development and spread of resistant bacteria [73].

Silage, feed production, and animal waste management can also contribute to the spread of ARG [74]. Slurry and wastewater from livestock operations can contaminate the environment (soils and water) and crops with antibiotic residues and ARB, potentially affecting human health (Figure 1) [73–75]. Addressing this issue demands a collaborative effort involving effective regulations, sustainable agricultural practices, and increased awareness among all stakeholders in the food chain.

### 3.1. Influence of Food Processing and Preservation Techniques

Bacterial survival and growth in food are influenced by processing techniques, preservation methods, and adherence to food safety practices [57]. Unlike industrial dairy processing, traditional PDO cheese dairies often avoid using additives and preservatives [1, 5]. The quality of Protected PDO cheeses is guaranteed by using high-quality raw materials, adhering to strict hygiene standards, and harnessing the benefits of natural fermentation [1, 21, 76].

During cheese production, milk proteins coagulate into curds upon the addition of rennet or other coagulants, followed by the draining of whey. The subsequent shaping and curing processes define whether the cheese will be semicured or fully cured, influencing its texture from soft to hard and affecting overall firmness [77]. During this process, cheese factory workers can introduce ARB poor hygiene practices, such as not washing hands or wearing contaminated clothing (Figure 1), also, inadequately cleaned equipment can spread ARB between production batches.

During the curing/maturation process, cheeses are kept under controlled conditions for varying periods, allowing for the development of distinctive biochemical, physicochemical, and organoleptic characteristics [77, 78]. The cheese is treated regularly to prevent mold growth, ensuring its quality and preservation. Improper handling of cheese or poor sanitation can result in the transfer of resistant bacteria from contaminated surfaces or workers to the cheese. The spread of foodborne pathogens and ARB in cheese by direct or/and cross-contamination during processing has been documented by several studies [79–81].

Organic acids, such as lactic, acetic, butyric, and sorbic acid, contribute to the low pH in certain cheeses, creating an environment that limits the survival of pathogenic bacteria [82, 83]. Food safety measures like refrigeration, pasteurization, and bio-protective cultures create additional barriers to microbial growth in cheese [82, 83].

Despite these measures, numerous cases of cheese-related illness outbreaks have been reported in Europe and other parts of the world in recent decades [77, 79]. Soft cheeses crafted from raw milk have commonly served as a vehicle for foodborne pathogens, although pasteurized-milk cheeses have also been implicated in outbreaks as carriers of the causative agents [77].

### 3.2. Influence of Biofilms

Biofilms are complex and highly structured aggregations of sessile bacteria, which are formed on biotic or abiotic surfaces, embedded in a self-produced extracellular matrix of exopolysaccharides, proteins, and DNA [40, 84–86]. Biofilms represent an important source of hazard in food industry and cause a significant health and economic impact [40, 87, 88]. Biofilms are implicated in recurrent contamination and outbreaks within food production environments [89]. Unlike antibiotic resistance, which involves genetic changes, persistence is a phenotypic adaptation that poses unique challenges for detection and control.

Several studies have demonstrated that microorganisms within biofilms exhibit significantly higher resistance to antimicrobial agents, often requiring 10 to 1000 times the concentration needed to eradicate equivalent planktonic populations [38, 39, 87, 88]. The high resistance observed in biofilms is attributed to quorum sensing, a microbial communication mechanism that enables coordinated responses to environmental challenges. Quorum sensing allows biofilm-dwelling microorganisms to conserve resources, reduce metabolic activity, and activate protective mechanisms against antimicrobial agents [84, 85].

The presence of biofilms in cheese factory environments poses a significant risk, as they can harbor a diverse microbial community, including beneficial LAB, spoilage organisms, and potentially pathogenic microorganisms [38, 40, 90]. Within biofilms, interactions between diverse microbial species facilitate the exchange of genetic material, enhancing the biofilm's evolutionary adaptability and resilience [38, 40, 91].

The microbial diversity of milk used for cheese production make cheese factory environments highly susceptible to biofilm formation increasing the risk of contamination of processed cheese by ARB, highlighting the importance of stringent preventive measures [5, 38, 87]. The protein- and fat-rich nature of the milk and dairy by-products used in cheese production creates an ideal environment for bacterial growth and biofilm formation, while the combination of high humidity levels and moderate temperatures commonly present in cheese factories further favors microbial proliferation [92]. Materials widely used in the process, such as stainless steel and plastic, are susceptible to biofilm formation, especially when their surfaces are damaged or scratched [38, 39].

Several metagenomic studies have revealed a high and diverse presence of genetic material related to antimicrobial resistance in cheese factories, attesting to the fact that dairies act as reservoirs for ARGs [5, 70, 93, 94]. These studies emphasize the importance of understanding and monitoring the spread of these genes in the context of food safety.

Yao et al. [95] evaluated the antibiotic resistance profile of *Lactococcus*, *Lactobacillus*, and *Streptococcus* isolated in cheese and found high resistance to sulfonamides (100%), aminoglycosides (91.7%), and tetracycline (31%). In *Enterococcus* recovered from raw milk cheese, high resistance was found to the antibiotic's vancomycin (87.5%), erythromycin (75%), tetracycline (50%), and penicillin (37.5%) [38]. Multidrug-resistant (MDR) diarrheagenic *E. coli* was recovered in Minas raw milk cheese in Minas Gerais [96]. A study of antibiotic resistance *Staphylococcus* species in a dairy factory showed multidrug resistance in 52% of the isolates, with resistance to penicillin being the most frequent, followed by cefoxitin, oxacillin, gentamicin, ciprofloxacin, and chloramphenicol [97]. Isolates recovered from cheese samples, packer equipment, cheese mold, and food handlers showed a similar antibiotic profile and were found to carry the mecA gene [97]. The findings reported in this study suggest that antibiotic resistance and highly virulent strains from different sources can be found in the dairy processing environment, causing significant concern for researchers, producers, and consumers.

### 3.3. Inadequate Sanitation Practices and Use of Disinfectants or Biocides

The overuse of disinfectants and biocides without proper protocols can facilitate the spread of antibiotic resistance through HGT [98]. Excessive or inappropriate use of certain biocides can also induce cross-resistance, where bacteria become resistant to both disinfectants and antibiotics [99, 100]. Some disinfectants, such as quaternary ammonium compounds or chlorine-based agents, can trigger genetic mutations or select for resistance mechanisms similar to those used against antibiotics [101]. For instance, efflux pumps, proteins that expel toxic compounds, can be activated by biocides and pump out antibiotics, reducing their effectiveness [102, 103]. Biocide-tolerant bacteria are more likely to exhibit multidrug resistance, posing a significant threat to food safety and public health [100].

When cleaning and sanitation procedures fail to completely remove bacterial contaminants from surfaces like conveyor belts, cutting tools, storage tanks, and pipelines, bacteria can survive and form biofilms [104, 105]. Biofilms provide additional protection against cleaning agents and disinfectants, making them persistent sources of contamination that can release bacteria into subsequent production batches.

Repeated exposure to sublethal doses of disinfectants can select for increasingly resistant strains, making the bacterial communities in the production environment more difficult to control [98]. This ongoing cycle of contamination and resistance can affect multiple product lines within the same facility, spreading ARB across different cheese varieties or dairy products [92, 106]. If resistant bacteria enter the final product, they can reach consumers, leading to foodborne illness outbreaks that are difficult to treat due to the bacteria's resistance to antibiotics.

### 3.4. Horizontal Gene Transfer

HGT refers to the transfer of genetic material, including ARGs, between organisms across different species or lineages [107]. The transfer of ARGs between bacteria can lead to the emergence of MDR strains, posing a serious threat to public health and food safety [108]. HGT is particularly concerning in cheese factories due to the high microbial diversity of cheese matrix that facility different mechanisms of HGT, namely, conjugation, transformation, and transduction [109].

In cheese factories, conjugation, a process involving the direct transfer of genetic material between two bacterial cells in physical contact, is the most important mechanism [109, 110]. Conjugation is the primary mechanism of HGT in *Enterococcus* species, significantly contributing to the spread of ARGs within *Enterococcus* communities in cheese-making environments [111, 112]. An example of this is vancomycin resistance in *E. faecium* and *E. faecalis* species, mediated by a conjugative plasmid [113].

Given the high concentration and diversity of bacteria in cheese, especially during fermentation, conjugation facilitates the rapid dissemination of genetic information [109]. In traditional cheeses made from raw milk, where the microbial load is high, the chances of conjugation occurring are increased, allowing antibiotic-resistant strains to spread rapidly across batches of cheese. The formation of biofilms on equipment surfaces in cheese-making factories also provides an ideal environment for conjugation [114].

Transformation, the uptake of free DNA from the environment by bacterial cells, is another mechanism by which Enterococcus species can acquire new genes in cheese-making facilities. There, bacteria take up naked DNA from their surroundings, which can come from dead bacterial cells or be released into the environment during processing [115]. This process can occur when bacterial cells are lysed during pasteurization, fermentation, or cleaning operations, releasing their DNA into the milk or cheese curd [116]. If ARGs or other virulence factors are present in the environment, Enterococcus species in the cheese-making process may acquire them through transformation. However, transformation is less frequent than conjugation and requires the bacteria to be in a “competent” state, capable of absorbing and integrating foreign DNA [57].

Transduction is another mechanism of gene transfer that occurs in *Enterococcus faecium* and *Enterococcus faecalis*. In transduction, bacteriophages (viruses that infect bacteria) transfer DNA between the bacteria [117]. Although transduction is less common than conjugation in *Enterococcus* species, it can still play a role in spreading ARGs or other virulence factors [111, 112, 117]. In cheese-making factories, where bacteriophages may be present in the milk or introduced through environmental contamination, transduction could contribute to the spread of unwanted traits in the microbial community [118]. Although phage contamination is generally less common than bacterial contamination, phages can persist in dairy environments and pose a risk in cheese production if they facilitate gene transfer between Enterococcus populations [118].

## 4. Risks Associated With *Enterococcus faecium* and *Enterococcus faecalis*

### 4.1. Benefits of *E. faecium* and *E. faecalis* in Cheese

*E. faecium* and *E. faecalis* are LAB that play a vital role in the fermentation and ripening of various traditional and nontraditional cheeses. Though they are often associated with gut microbiota and probiotics [119], their contribution to cheesemaking has garnered attention due to the beneficial traits they bring to the production process [120, 121]. These bacteria are commonly found in raw milk and are added intentionally as starter or adjunct cultures in some cheese varieties [38, 120]. Their ability to thrive in extreme conditions, such as high salt concentrations, low pH levels, and elevated temperatures, makes them particularly well suited for cheese fermentation.

One of the key benefits of *E. faecium* and *E. faecalis* in cheesemaking is their contribution to the development of flavor. These bacteria produce enzymes that break down proteins, fats, and carbohydrates in the cheese matrix, resulting in the release of a wide array of volatile compounds that give cheeses their distinctive taste and aroma. In particular, the breakdown of casein by Enterococcus strains enhances the development of complex flavors in both hard and soft cheeses. In traditional Mediterranean cheeses like Pecorino, Feta, and various artisanal goat and sheep cheeses, these strains are naturally present and significantly influence the tangy, sharp, or savory characteristics of the final product. Moreover, these Enterococcus species contribute to texture improvement, particularly in soft cheeses like Ricotta or Mozzarella. Their proteolytic activity helps in breaking down milk proteins, leading to a smooth and creamy consistency. In nontraditional or experimental cheeses, where novel microbial combinations are explored, *E. faecium* and *E. faecalis* have been used to enrich both flavor and texture, offering new possibilities for cheesemakers looking to create innovative products.

### 4.2. Risks of *E. faecium* and *E. faecalis* in Cheese

Despite the importance of *E. faecium and E. faecalis* as starter and probiotic strains, they also present specific risks that both cheesemakers and consumers should be aware of, especially in the production and consumption of traditional and nontraditional cheeses. One of the primary risks associated with *E. faecium* and *E. faecalis* in cheese production is the potential to spread of ARGs, particularly vancomycin-resistant *Enterococcus* (VRE) [38]. The raw sheep's milk cheese production chain harbors a potential concern, *E. faecalis* and *E. faecium*, two seemingly harmless bacteria, can serve as silent carriers of ARGs within this industry. Understanding their role in this context is critical to ensuring the safety and sustainability of raw milk cheese production. Several factors in the production process create conditions that facilitate the spread of ARGs. The absence of pasteurization, a key step in eliminating harmful bacteria, allows *E. faecalis* and *E. faecium* to thrive. Additionally, the use of antibiotics in animal husbandry can spill over into the milk, further increasing the risk of resistant bacteria being present. Furthermore, the diverse microbial communities present during cheesemaking can facilitate the horizontal transfer of resistance genes between different bacterial species.

Enterococci are notorious for their ability to harbor diverse mobile genetic elements within their genomes [121]. These elements, such as plasmids, transposons, prophages, and insertion sequences, can be readily integrated and utilized by enterococci [118, 122]. This facilitates the efficient transfer of acquired determinants, including virulence factors and ARGs, among strains of the same species, or even between species within the same genus or beyond. Notably, many of these highly transmissible plasmids are known to carry genes associated with enterococcal virulence and antibiotic resistance [121]. Virulence traits and ARGs in enterococci were previously reported to be caused by gene horizontal or vertical transfer mechanisms and by the ability to receive genetic material [121, 123]. Experimental studies have confirmed the horizontal transfer of ARGs, such as *ermB*, from an enterococcal strain of animal origin to a strain of human origin [121]. This mechanism, facilitated by the transfer of genetic elements such as plasmids or transposons, plays a more significant role in the dissemination of antimicrobial resistance than the clonal spread of ARB [121, 124, 125]. A significant concern is the potential for trans-conjugation, a process through which enterococci can acquire virulence and antibiotic resistance determinants. This poses a serious threat to the safety of enterococcal strains that currently lack these harmful genes, as they could acquire them from both human and non-human reservoirs [121]. This raises significant concerns regarding the safety of using such strains as probiotics.

While the allure of raw sheep's milk cheese is undeniable, it is crucial to address the potential public health concerns linked to antibiotic resistance. Understanding the roles of *E. faecalis* and *E. faecium*, adopting responsible production practices, and ensuring continuous monitoring are essential steps to safeguard the safety and sustainability of this cherished tradition. These measures protect both consumers and the broader integrity of our food system. Notably, *E. faecalis* and *E. faecium* have been associated with a high potential for the horizontal transfer of ARGs, virulence factors, and elements that promote biofilm formation on various surfaces, including stainless steel, polyvinyl chloride, and polystyrene [23, 24, 38, 40, 126].

Several studies have investigated the antibiotic resistance profiles of *Enterococcus* strains isolated from cheese [1, 4, 23, 38, 126–131]. One such study focused on *Enterococcus* strains recovered from raw ewe's milk [38], revealing significant resistance levels: 75% of isolates were resistant to erythromycin, 50% to tetracycline, and 87.5% to vancomycin. Additionally, all VRE isolates exhibited multidrug resistance and harbored the *vanA* gene [38]. Similar results were found in *Enterococci* isolated from ewe's and goat's milk cheeses, where *E. faecium* exhibited 100% resistance to vancomycin, while *E. faecalis* demonstrated 85.7% resistance to vancomycin and 71.4% resistance to erythromycin [128].

In *Enterococcus* species, *vanA* is one of the key genes regulating and expressing vancomycin resistance. This gene, along with other vancomycin resistance–related genes (*vanR*, *vanS*, *vanH*, *vanX*, and *vanZ*), is located on the transposon Tn1546, which is frequently associated with plasmids in *E. faecium* [132]. The expression of these genes leads to the production of altered peptidoglycan precursors ending in D-Ala–D-lactate instead of the typical D-Ala–D-Ala structure [132, 133]. Because *vanA* is plasmid-mediated, vancomycin resistance in *E. faecium* is likely the result of HGT, making it transferable to other bacteria, either within the same species or across different species. Similarly, erythromycin resistance in *Enterococcus* can also spread via HGT. This resistance is linked to the presence of *erm* genes (*ermA*, *ermB*, and *ermC*), which encode erythromycin ribosome methylases [134]. These genes were initially identified on the Tn554 transposon in the chromosome of *Staphylococcus aureus* [135].

Tetracycline resistance was detected in 75% of *E. faecalis* and 25% of *E. faecium* isolates from Serra da Estrela PDO cheese [38], as well as in 75% of *E. faecalis* and 25% of *E. faecium* isolates from Azeitão and Nisa cheeses [1]. In Serra da Estrela PDO cheese, all tetracycline-resistant phenotypes were associated with the presence of the *tetM* gene [38]. This gene, which is highly prevalent among *Enterococcus* species, is primarily located on the bacterial chromosome and is often linked to conjugative transposons belonging to the Tn916/Tn1545 family [136].

### 4.3. Mitigation of the Risk

The results from this study highlight the significant risk posed by ARGs and ARB strains in the dairy environment, especially in the context of *Enterococci* species such as *E. faecium* and *E. faecalis* in cheese production. The findings raise important concerns for researchers, producers, and consumers alike regarding the potential health implications of antibiotic resistance in the food chain.

To mitigate these risks, several strategies to reduce antibiotic resistance in cheese production focus on controlling antibiotic use and ensuring safety throughout the production process should be implemented. Limiting antibiotic use on dairy farms to only essential treatments, as prescribed by veterinarians, and avoiding antibiotics critical for human health reduce the risk of developing ARB. Regularly testing milk for antibiotic residues before cheese production helps identify and prevent contamination, ensuring that antibiotics do not reach consumers. Implementing strict hygiene protocols in the milking, transport, and cheese production areas as well as proper sanitation minimizes the spread of bacteria, reducing opportunities for antibiotic-resistant strains to multiply.

The study also underscores the importance of understanding gene transfer mechanisms in *Enterococci*, particularly in cheese-making facilities. Gene transfer via conjugation plays a dominant role in the spread of ARGs, with transduction and transformation acting as supplementary pathways. These processes are especially prevalent in high-contact environments, such as biofilms on processing equipment or in raw-milk cheeses. Research into these mechanisms is crucial for developing strategies to enhance the safety of both traditional and industrial cheese varieties.

## 5. Conclusion

The study highlights that ARGs and ARB in dairy processing environments pose significant risks to food safety. Key findings indicate that *Enterococci*, particularly *E. faecium* and *E. faecalis*, contribute to the spread of ARGs through conjugation, transduction, and transformation, especially in biofilms and raw-milk cheeses. To mitigate these risks, the study recommends responsible practices such as regular monitoring for ARB, strict hygiene and sanitation protocols, cautious antibiotic use in dairy farming, and strategies to prevent biofilm formation. These measures are essential to ensure the safety and sustainability of cheese production, safeguarding both consumer health and global food systems.

## Figures and Tables

**Table 1:** Profiles and antibiotic resistance genes identified in fermented foods.

Microorganisms	Type of fermented food	Antibiotic resistance profile	Antibiotic resistance genes	Reference
*Lactobacillus pentosus*	Aloreña green table olives	STR, VAN, TEI, TRI, STX, CEF	*NorA*, *MepA* and *MdeA*	Casado Muñoz et al. [41]
*Leuconostoc pseudomesenteroides*				

*Lactobacillus helveticus*	Fermented milk	VAN, CIP, TET	*vanX*, *vanE*, *gyr(A)*, and *tetM*	Guo et al. [43]
	Chinese sausages and vegetables	TET, ERY, CIP, CHL, KAN, AMP, CLI	*tetM*, *ermB*, *aphA3*, *mefA*	Pan, Hu, and Wang [42]

*Lactobacillus casei*	Milk	VAN, CIP, TET	*vanX*, *vanE*, *gyr(A)*, *tetM*	Guo et al. [43]
	Turkish cheese, yogurt, kefir, and boza	CHL, TET, ERY, CIP, VAN	*tetM*, *vanA*	Basbülbül, Özteber, and Biyik [44]
	Chinese fermented milk	STR, GEN, KAN, CIP, CHL, VAN, TET	*tetM*, *sul1*, *sul2*, *strA*, *strB*, *aac(6*′*)-aph(2*″), *aph(3*″*)-II* and *aph(3*″*)-III*	Li et al. [45]

*Lactobacillus plantarum*	Fermented milk	VAN, CIP, TET	*vanX*, *vanE*, *gyr(A)*, and *tetM*	Guo et al. [43]
	Chinese sausages and vegetables	TET, ERY, CIP, CHL, KAN, AMP, CLI	*tetM*, *ermB*, *aphA3*, *mefA*	Pan, Hu, and Wang [42]
	Turkish cheese, yogurt, kefir, and boza	CHL, TET, ERY, CIP, VAN	*tetM*, *vanA*	Basbülbül, Özteber, and Biyik [44]
	Tibetan kefir grains	TET, ERY, CLI, CHL	*aac(3)*, *lsa*, *tetM*, *TetL*, *tetW*, *ErmB*	Zheng et al. [46]

*Lactobacillus bulgaricus*	Chinese yogurts	PEN, KAN	*tetM*, *ant(6)*, *aph(3″)-IIIa*	Zhou et al. [47]
	Chinese yogurts	VAN, GEN, STR	*Van*, *aadB*, *aph*, *aadA2*	Wang et al. [48]
	Traditional fermented milk	KAN, CIP, STR, TRI, AMP, VAN	*rpoB*, *ermB*, *aadA*, *blaZ*, *cat*, *vanX*	Guo et al. [49]
	Chinese milk	STR, GEN, KAN, CIP, CHL, VAN TET	*tetM*, *sul1*, *sul2*, *strA*, *strB*, *aac(6*′*)-aph(2*″), *aph(3*″*)-II*, *aph(3*″*)-III*	Li et al. [45]

*Lactococcus lactis*	Polish raw milk and artisanal products	TET	*tetM*, *tetS*	Zycka-Krzesinska et al. [50]
	Turkish cheese, yogurt, kefir, and boza	CHL, TET, ERY, CIP, VAN	*tetM*, *vanA*	Basbülbül, Özteber, and Biyik [44]

*Lactobacillus paracasei*	Raw Ewe's milk cheese, raw water buffalo cheese, and raw cow milk cheese	TET, ERY	*tetW*, *tetM*, *ermB*	Comunian et al. [51]
	Cultures for use in the food industry	TET, KAN, CHL	*tetM*, *tetW*, *tetO*, *blaOXA*, *blaZ*, *cat*	Zarzecka, Chajęcka-Wierzchowska, and Zadernowska [52]
	Chinese fermented milk	STR, GEN, KAN, CIP, CHL, VAN, TET	*tetM*, *sul1*, *sul2*, *strA*, *strB*	Li et al. [45]
	Chinese dairy products	CHL, VAN, STX, TET, GEN, ERY, CLI	*Not available*	Xu et al. [53]

*Lactobacillus paraplantarum*	Cultures for use in the food industry	TET, KAN, CHL	*tetM*, *tetW* and *tetO*, *blaZ*, *cat*	Zarzecka, Chajęcka-Wierzchowska, and Zadernowska [52]
*Lactobacillus delbrueckii*	Cultures for use in the food industry	TET, KAN, CHL	*tetM*, *tetW* and *tetO*, *blaZ*, *cat*	
	Chinese dairy products	CHL, VAN, TRI, TET, CFT, GEN, ERY, CLI	*Not available*	Xu et al. [53]

*Lactobacillus brevis*	Chinese fermented sausages and vegetables	TET, ERY, CIP, CHL, KAN, AMP, CLI	*tetM*, *ermB*, *aphA3*, *mefA*	Pan, Hu, and Wang [42]

*Lactobacillus kefiri*	Tibetan kefir grains	TET, ERY, CLI, CHL	*aac(3)*, *lsa*, *tetM*, *tetL*, *tetW*, *ErmB*	Zheng et al. [46]

*Streptococcus thermophilus*	Chinese yogurts	VAN, GEN, STR	*van*, *aadB*, *aph*, *aadA2*	Wang et al. [48]

*Lactobacillus fermentium*	Chinese fermented sausages and vegetables	TET, ERY, CIP, CHL, KAN, AMP, CLI	*tetM*, *ermB*, *aphA3*, *mefA*	Pan, Hu, and Wang [42]

*Enterococcus faecium*	Chinese fermented sausages and vegetables	TET, ERY, CIP, CHL, KAN, AMP, CLI	*tetM*, *ermB*, *aphA3*, *mefA*	Pan, Hu, and Wang [42]
	Turkish cheese, yogurt, kefir, and boza	CHL, TET, ERY, CIP, VAN	*tetM*, *vanA*	Basbülbül, Özteber, and Biyik [44]
	Pasteurized fermented dairy products	ERY, TET, AMP, OXA, AZT, VAN	*ermB*, *ermC*, *vanC1*, *vanC2*, *vanC3*	Mariam [54]
	Pasteurized fermented dairy products	ERY, TET, AMP, OXA, CFT, AZT, VAN	*ermB*, *ermC*, *vanC1*, *vanC2*, *vanC3*	Mariam [54]
	Raw milk cheese	TET, ERY, VAN, GEN, P, RD	*ermA*, *ermC*, *tetM*, *vanA*, *pbp5*, *blaZ*	Salamandane et al. [38]

*Enterococcus faecalis*	Raw milk cheese	P, TET, RD, ERY, VAN	*ermC*, *tetM*, *vanA*, *vanB*, *blaZ*	Salamandane et al. [38]

*Lactobacillus namurensis*	Chinese fermented sausages and vegetables	TET, ERY, CIP, CHL, KAN, AMP, CLI	*tetM*, *ermB*, *aphA3*, *mefA*	Pan, Hu, and Wang [42]

*Lactobacillus fermentum*	Milk products	ERY, TET	*tetM*, *tet(K)*, *erm(C)*	Anisimova and Yarullina [55]
	Turkish cheese, yogurt, kefir, and boza	CHL, TET, ERY, CIP, VAN	*tetM*, *vanA*	Basbülbül, Özteber, and Biyik [44]
	Chinese dairy products	CHL, VAN, CFT, TRI, TET, GEN, ERY, CLI	*Not available*	Xu et al. [53]

*Enterococcus species*	Artisanal dairy products	VAN, AMP, TET, STR, GEN, KAN, ERY, CHL, CLI, CIP	*vanA*, *vanB*, *vanC*, *blaZ*, *tetL*, *tetK*, *aacA-aphD*, *aadE*, *ermA*	Amidi-Fazli and Hanifian [24]
	Raw milk cheese	AMP, CIP, LEV, LZD, QD, TEC, TET, VAN	Not available	Bastião Rocha et al. [1]

*Lactobacillus coryniformis*	Turkish cheese, yogurt, kefir, and boza	CHL, TET, ERY, CIP, VAN	*tetM*, *vanA*	Basbülbül, Özteber, and Biyik [44]

*Streptococcus thermophilus*	Chinese fermented milk	STR, GEN, KAN, CIP, CHL, VAN, TET	*tetM*, *sul1*, *sul2*, *strA*, *strB,*	Li et al. [45]
	Pasteurized fermented dairy products	ERY, TET, AMP, OXA, CFT, AZT, VAN	*ermB*, *ermC*, *vanC1*, *vanC2*, *vanC3*	Mariam [54]

*Lactobacillus acidophilus*	Chinese dairy products	CHL, VAN, CFT, TRI, TET, GEN, ERY, CLI	*Not available*	Xu et al. [53]
	Chinese milk	STR, GEN, KAN, CIP, CHL, VAN, TET	*tetM*, *sul1*, *sul2*, *strA*, *strB*, *aac(6*′*)-aph(2*″), *aph(3*″*)-II* and *aph(3*″*)-III*	Li et al. [45]

*Lactobacillus rhamnosus*	Chinese milk	STR, GEN, KAN, CIP, CHL, VAN, TET	*tetM*, *sul1*, *sul2*, *strA*, *strB*, *aac(6*′*)-aph(2*″), *aph(3*″*)-II* and *aph(3*″*)-III*	Li et al. [45]
	Chinese dairy products	CHL, VAN, CFT, TRI, TET, GEN, ERY, CLI	Not available	Xu et al. [53]

Abbreviations: AMP, Ampicillin; AZT, Azithromycin; CEF, Cefuroxime; CFT, Ceftriaxone; CHL, Chloramphenicol; CIP, Ciprofloxacin; CLI, Clindamycin; ERY, Erythromycin; GEN, Gentamycin; KAN, kanamycin; LEV, levofloxacin; LZD, linezolid; OXA, Oxacillin; P, penicillin; PEN, Penicillin; QD, quinupristin-dalfopristin; RD, rifampicin; STR, Streptomycin; STX, TRI/Sulfamethoxazol; TEC, teicoplanin; TEI, Teicoplanin; TET, Tetracycline; TRI-Trimethoprim/sulfamethoxazole; VAN, Vancomycin.

## Data Availability

Data sharing is not applicable to this article as no datasets were generated or analyzed during the current study.
